# Applying Monte Carlo Method for Straight-Line Model Sensor Calibration

**DOI:** 10.3390/s26092907

**Published:** 2026-05-06

**Authors:** Pedro M. Ramos, Fernando M. Janeiro

**Affiliations:** 1 Instituto de Telecomunicações, Instituto Superior Técnico, Universidade de Lisboa, Av. Rovisco Pais 1, 1049-001 Lisboa, Portugal; 2 Instituto de Telecomunicações, Escola de Ciências e Tecnologia, Universidade de Évora, Largo dos Colegiais 2, 7004-516 Évora, Portugal

**Keywords:** calibration, sensor straight-line model, measurement uncertainty, weighted total least squares regression, Monte Carlo method, uncertainty evaluation

## Abstract

Sensors are used in measurement systems to enable estimation of physical parameters. Their calibration is an essential requirement and to perform the overall system/sensor calibration, its input is changed while the output is measured. Parameters from an appropriate sensor model are then determined from these measurements. If the model is a straight-line, a first-order least squares linear regression is commonly used to estimate the slope and offset—this is often called simple linear regression. However, this method is unable to consider uncertainty in the sensor/system input measurements. This paper reviews the possible methods to estimate the optimal straight-line parameters considering uncertainties in both input and output measurements. The Monte Carlo Method can deal with all types of uncertainties in each of the measurements, whether sensor inputs or outputs, and also take into account possible covariances of these measurements. A key aspect of this work is the application to the heteroscedastic case, where measurement uncertainties vary across observations. An MCM-based strategy is proposed to optimize the selection of new measurement input values to minimize the estimated slope uncertainty. This strategy is shown to significantly reduce, in the presented case, the number of required measurement values.

## 1. Introduction

Sensors have become ubiquitous, as they are extensively used in industrial systems, space exploration, research and development facilities, transportation vehicles, robotic systems, household appliances and personal devices. They are the cornerstone behind Wireless Sensor Networks (WSNs), the Internet of Things (IoT), cyber-physical systems and Industry 4.0. As the world continues to get even more connected, sensors are the key element that enables system interconnection with the real physical world. Nearly all systems/devices are equipped with sensors, with many including tens or hundreds of sensors, that measure and monitor different parameters, which are then handled in microcontroller or processor units.

Most sensors transform the physical quantity to be measured into an electrical quantity, typically a current or a voltage, which can then be converted into the digital domain and processed. For the processor unit to estimate the physical quantity, it needs to know the sensor transfer function and therefore it must be properly calibrated before use. This calibration procedure is crucial, as it determines key sensor aspects, including static and dynamic parameters, and the overall sensor quality is evaluated based on these characteristics. The usefulness of sensors is dependent on parameters such as sensitivity, responsiveness, non-linearity, drift, hysteresis and bias among others [1]. It is also common and useful for the measurement system to include, in its measuring chain, some analog signal conditioning before digitalization. In this case, the calibration procedure can be applied just to the sensor or to the complete measuring chain. Within the context of this work, the term sensor calibration is used, but it can, without loss of generalization, be applied to the complete measuring system.

The VIM [2] defines, in paragraph 4.31, the calibration curve as the “*expression of the relation between indication and corresponding measured quantity value*” where “*measured quantity value*” can be replaced by “*measured value*”. Sensors included in measurement systems require the determination of this calibration curve. The calibration process consists of supplying the sensor with a set of inputs and measuring the corresponding outputs to obtain, for an appropriate model, the calibration curve. This process is necessary to minimize systematic measurement errors and to enable the estimation of the physical quantity that the sensor is measuring. The determination of the sensor calibration curve is essential and its optimization is fundamental in metrology.

In [3], a set of methodologies to calibrate sensors with statistical estimation algorithms is presented and examples are included. The process is divided into three steps: (i) description of the sensing process with a statistical model; (ii) estimation of the model parameters; (iii) processing new measurements to improve the statistics of the estimated parameters.

The optimum selection of the measurement points for the calibration process was analyzed in [4] for linear, quadratic, cubic and fourth-order input/output sensor relationships. Therein, it is concluded that, for zero-mean normally distributed variables with the same standard deviation: (i) the optimum location of the sensor inputs does not correspond to equally spaced values in the input range; (ii) an increase in the number of measurements reduces the calibration coefficients uncertainty; (iii) the standard deviations of the calibration parameters decrease with increased repetitions of the same sensor inputs, but there is no improvement by indefinitely continuing to add repetitions.

A discussion on the experimental design techniques to optimize the calibration process was published in [5], where a relative humidity sensor and a heat meter were considered. The process is based on the since-deprecated error propagation method suggested in the 1993 version of the Guide for the Expression of Uncertainty (GUM) [6], which has been replaced with the law of propagation of uncertainty in updated versions of the GUM [7].

An A-optimality criterion is used in [8] to select the ideal measurement points for calibration. The method applicability is demonstrated for the calibration of a differential pressure gauge using a classical Least Squares (LS) method for a second-order polynomial input/output relation. The considered measurement errors are zero-mean normal/Gaussian distributed.

An example based on relative humidity (RH) resistive and capacitive sensors using different saturated salt solutions is used to determine the ideal measurement/calibration points in [9]. Uncertainty propagation is used to determine the relative humidity uncertainty. Different order polynomials were evaluated using the residual plots together with the *t*-value of the highest-order parameters.

In the situations where the sensor input/output relation is a straight-line, a first-order linear regression is commonly used to estimate the offset (or intercept) and slope parameters that define the straight-line analytical equation. Least squares, which was initially used for the estimation of the orbits of comets, was independently developed by Legendre [10] and Gauss in the early 1800’s. It is widely used in almost all research fields and taught to high-school and undergraduate students. The method minimizes the sum of the squares of the residuals between the measured outputs and the regression estimates. For sensor calibration, the LS method requires a set of sensor input and output values. The parametric values of the straight-line that best fits the set of measured values given the known sensor inputs are estimated by the method. Straight-line linear regression can also be used to determine the linearity or nonlinearity of a sensor, system, or device. However, the method neglects the measurement uncertainties of the input quantities and, in real calibration scenarios, both the input and output quantities have measurement uncertainties.

The technical specification ISO/TS 28037 [11] describes different straight-line calibration situations. It specifies how to estimate the slope and offset uncertainties as defined in the GUM. However, as explained in the note of paragraph 5.5.1, when the uncertainty propagation approximation is invalid, the propagation of distributions based on the Monte Carlo Method (MCM) should be used. The process of applying the MCM is beyond the scope of the technical specification, and therefore is not covered in the ISO/TS 28037 technical specification. A detailed analysis, presented in [12], suggests adjustments to the technical specification as, in some cases, it underestimates the calibration coefficient uncertainties. Although it only considers simulation situations and multivariate normal/Gaussian distributions, it also concludes that other assumptions will result in different outcomes and should be evaluated on a case-by-case basis. To cover all cases, it is stated that MCM should be used and its results are the reference for all situations.

MCM was used for straight-line linear calibration in the field of dimensional metrology of a 1-D measuring machine in [13]. The measurement uncertainty was considered only for the dependent variable as the input uncertainties were disregarded. The Probability Density Function (PDF) of the measured values was considered to be Gaussian/normal and its uncertainty was the same for all the measurement values, i.e., the homoscedastic situation. The MCM estimated the slope and offset average values, standard deviations and their covariance.

This paper discusses in detail the use of MCM for estimation of the straight-line sensor calibration coefficients. It is capable of addressing any situation, including different PDFs for any of the measurement values (system/sensor input or output). It also accounts for covariances between measurements. Furthermore, it effectively handles heteroscedastic conditions that arise intrinsically from the measurements, for example due to the use of different instrument ranges leading to different uncertainty levels. This work is an extension of the preliminary study presented in [14], with a new experimental measurement setup and more in-depth discussion/analysis. Also added is the estimation of the output correlation in two extreme cases: one with only two measurements, and the other with a very large number of measurements. Additional discussion and analysis is included for the comparison of the MCM results with the ones obtained from the weighted total least squares algorithm that can also take into account sensor/system input uncertainties.

Since the time and cost of a calibration procedure depends on the number of measured calibration points, an MCM simulation-based strategy for the choice of the location of these points is proposed. This strategy avoids measuring points at non-ideal locations, and allows the optimization of the uncertainty of the estimated sensor parameters. The work developed in this paper contributes to filling the gaps in the ISO/TS 28037 technical specification by presenting and analyzing the performance of MCM in realistic straight-line sensor calibration scenarios. It considers different uncertainty PDFs for each input and output measurement values, which are not covered in the technical specification.

In Section 2, different straight-line sensor calibration algorithms are described together with their strengths and weaknesses, and the application of the Monte Carlo method is also presented. Section 3 describes the measurement setup used to retrieve the calibration measurements of a power grid voltage sensor-box, which are used throughout the remainder of this paper. The results obtained in different conditions are presented in Section 4 with discussion and analysis of the multiple situations considered. Section 5 proposes the selection strategy for the calibration measurement points. The conclusions are summarized in Section 6.

## 2. Straight-Line Sensor Calibration

To calibrate a sensor, a finite set (*N*) of measurement pairs are performed. Each pair includes a sensor input measurement, $x_{i}$, and a corresponding output measurement, $y_{i}$:

(1) $$\begin{array}{c} \left(x_{1},y_{1}\right)\\ \left(x_{2},y_{2}\right)\\ \cdots \\ (x_{N},y_{N}). \end{array}$$

For each of the measurements there is a corresponding uncertainty $u(x_{i})$ for the sensor input measurement, and $u(y_{i})$ for the sensor output measurement.

The determination of the sensor input/output relation is done using an algorithm that, based on the set of measured pairs, determines the coefficients of the equation/model that describes the sensor input/output relation.

Curve fitting regressions are divided into linear and nonlinear regressions. In linear regression models [15], which include, for example, the basic straight-line and polynomial fitting, the sensor output is modelled by the sum of a set of *K* finite terms whose coefficients $a_{k}$ are multiplied by known functions $f_{k}$ of the input *x*:

(2) $$y\left(x\right)=\sum_{k=1}^{K}a_{k}f_{k}\left(x\right).$$

When a linear regression is unable to model the input/output relation as defined in (2), a nonlinear regression is required—this case is not considered here but the Monte Carlo method could also be used in those situations.

In this paper, the input/output relation of the sensor is considered to be modeled by a straight-line (in some of the literature, called simple linear regression):

(3) y=b+mx

where *m* and *b* are the straight-line slope and offset, respectively. This is a particular case of (2) obtained with K=2, $a_{1}=b$, $f_{1}\left(x\right)=1$, $a_{2}=m$ and $f_{2}\left(x\right)=x$. The objective of the sensor calibration procedure is the determination of the coefficients *m* and *b* that best describe the sensor input/output relation.

**Figure 1:**
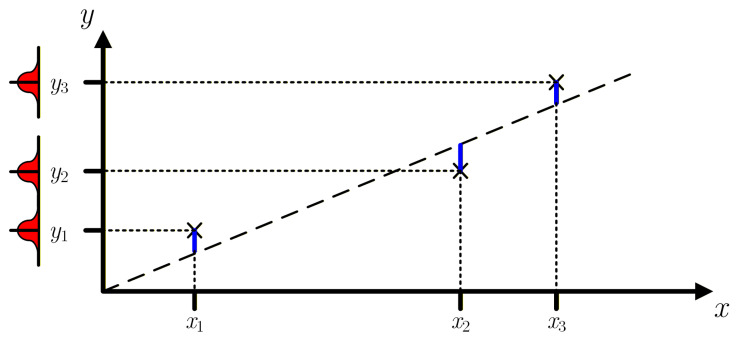
Graphical example of the least squares linear regression method for three measurement pairs (N=3). The blue lines represent the vertical distances between the measured values $y_{i}$ and the best fit straight-line (dashed black line). This ideal straight-line is drawn from the *m* and *b* coefficients obtained by minimizing the sum of the squared distances.

In Least Squares (LS) linear regression algorithms, the sum of the squared errors between the sensor outputs and the straight-line values at the corresponding input values $x_{i}$ is minimized. A simplified graphical representation of this method is shown in Figure 1. Since the errors/distances are measured vertically, this method does not take into account uncertainties of the measured sensor inputs, i.e., for all measurements it assumes $u(x_{i})=0$ and the only uncertainties considered are those of the sensor output measurements $u(y_{i})\neq 0$.

**Figure 2:**
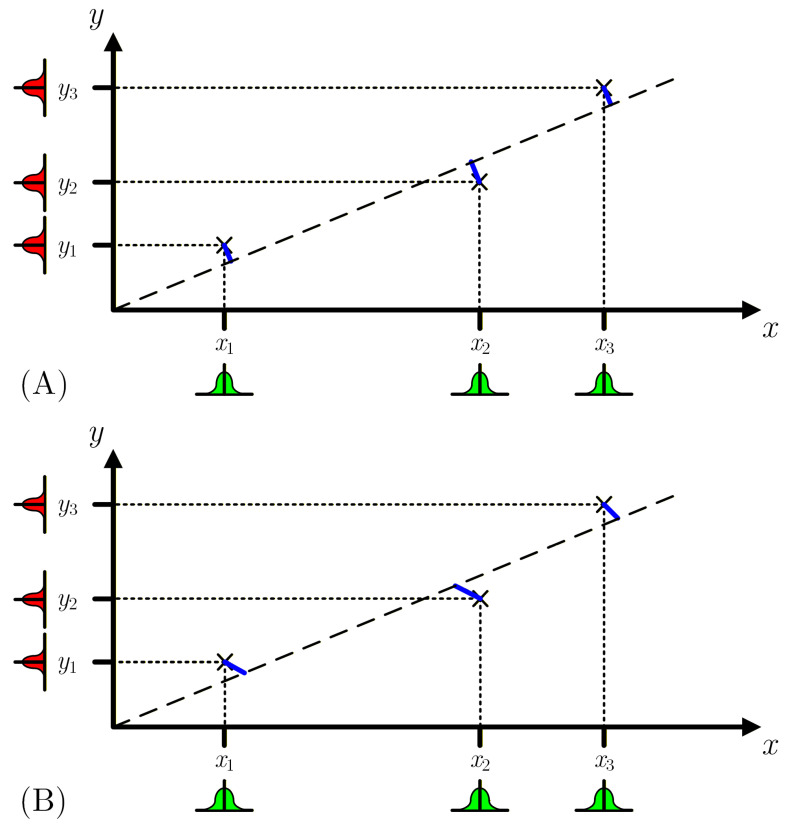
Representation of linear regression using Total Least Squares (TLS) in (**A**) and Deming regression in (**B**). The distances between the measured $y_{i}$ values and the best fit straight-line (dashed line) are represented by the blue lines. In the TLS, the input and output measurement uncertainties are the same, while in the Deming regression there is a ratio between the input/output uncertainties. In the TLS, the blue lines are perpendicular to the best fit line, while for the Deming regression the relative uncertainty of the inputs/outputs can drive the algorithm to favor one direction over the other.

The Total Least Squares (TLS) method, which is also called orthogonal linear regression, minimizes the sum of the perpendicular distances between the measured points and the straight-line [16], as represented in Figure 2A. It takes into account uncertainties in the sensor input measurements. However, this method is also limited, as it considers that all measurements, either sensor inputs or outputs, have the same uncertainty values—the PDFs represented in Figure 2A near $x_{i}$ and $y_{i}$ all have the same standard deviation.

Also capable of considering uncertainty in the sensor input measurements, the Deming regression [17] requires that the measurement uncertainties in both the input and output quantities are independent, have a normal distribution, are homoscedastic, and that there is a known common ratio of their variances as depicted in Figure 2B, i.e., $u\left(x_{i}\right)=\alpha \;u\left(y_{i}\right)$, where α is the relation between sensor input and output measurement uncertainties. However, due to the unique uncertainties of each measurement, which are usually dependent on the measured value and on the used instrument range, these conditions are often unverified.

To consider different uncertainties for each $x_{i}$ and $y_{i}$ without restrictions, an option is the Weighted Total Least Squares (WTLS) regression described in paragraph 7 of [11]. This method is also known as the weighted Orthogonal Distance Regression (ODR), Generalized Distance Regression (GDR), or errors-in-variables model. It assigns different weights, $w(x_{i})$ and $w(y_{i})$, to the orthogonal distances in their contribution to the cost function, which is iteratively minimized. The weight given to each measurement is the inverse of their standard uncertainty, $w\left(x_{i}\right)=1/u\left(x_{i}\right)$ and $w\left(y_{i}\right)=1/u\left(y_{i}\right)$. With these weights, measurements with lower uncertainty are given more relevance, i.e., its orthogonal distance to the straight-line is more penalized. One limitation of this method is that it is unable to consider covariances from the measured data. One method that can include these covariances, is the Generalized Gauss Markov Regression (GGMR), described in paragraph 10 of [11]. However, as the other methods described in [11], its uncertainty estimation is based on the propagation of uncertainties.

**Figure 3:**
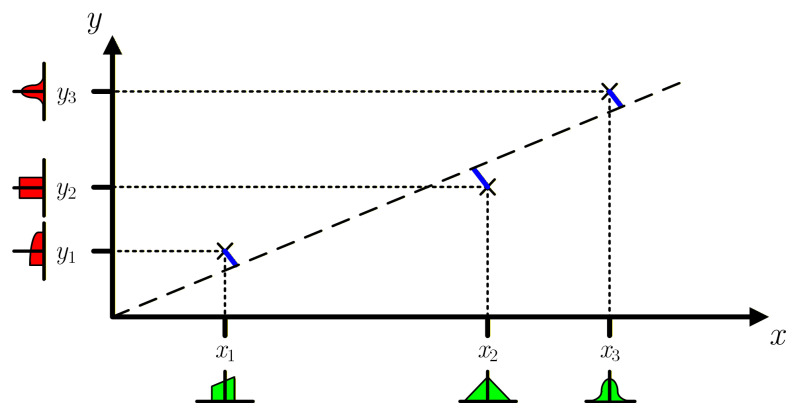
Graphical example of the Monte Carlo method (MCM). As in Figure 2, the blue lines represent the minimum distances between the measured $y_{i}$ values and the best fit straight-line considering weights defined by the inverse of the standard deviations of each measured value. The MCM can consider different uncertainties and different PDFs for each measured $x_{i}$ and $y_{i}$ value as shown.

To consider differently shaped PDFs and the covariances of the measurements (both inputs and outputs), the propagation of the distributions based on the Monte Carlo method (MCM) [18,19] must be used. This method randomly generates values of the sensor input and output based on the measurements according to their PDFs/covariances. It then estimates the model outputs, which in this case are the regression parameters slope *m* and offset *b* obtained using WTLS, and the process is repeated *M* times. Measurement covariances can be taken into account when generating the random values associated with the measurements. A statistical analysis of the estimated parameters can be used to assess their PDF and CDF (Cumulative Distribution Function) shapes, average values, standard deviations, correlation and uncertainties. The number of repetitions should be adjusted to achieve convergence, as described in paragraph 7.2 of [18]. A significant advantage of the MCM approach is that it allows the inclusion of heteroscedasticity situations, as is the case when the individual measurement uncertainties are different, as they depend on the actual measured values and instrument ranges, which is common in most instruments. In addition, it can consider any distribution (PDF) associated with the measurement uncertainties, as exemplified in Figure 3.

## 3. Experimental Setup

**Figure 4:**
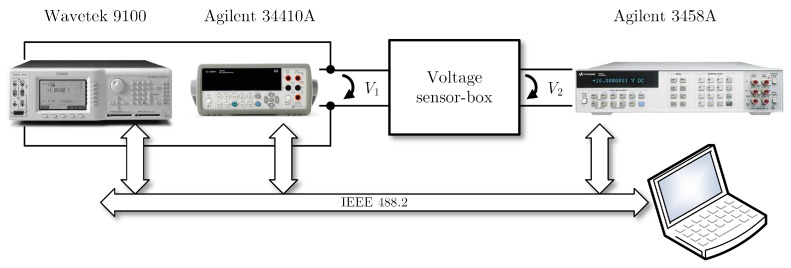
Measurement setup for the calibration of a LEM LV 25-P-based voltage sensor-box. The Wavetek 9100 calibrator supplies the input voltage at 60 Hz with voltages up to 300 V. The Agilent 34410A multimeter measures the sensor-box input voltage ($V_{1}$) and an Agilent 3458A multimeter measures the sensor-box output voltage ($V_{2}$). All instruments are controlled by IEEE 488.2 to set the input voltage and measure the input and output voltages.

The experimental setup is represented in Figure 4. The sensor-box is based on an LEM LV 25-P Hall-effect voltage sensor (LEM, Geneva, Switzerland) [20], configured for operation in the European nominal 230 V RMS power grid voltage. The sensor input voltage is supplied by a Wavetek 9100 calibrator (Wavetek, Hsinchu Science Park, Taiwan) [21] controlled by IEEE 488.2 that sequentially and automatically changes the sensor-box input voltage. To avoid interferences with the 50 Hz power grid, the AC source frequency is set to 60 Hz. The sensor-box input voltage is measured with an Agilent 34410A (Agilent Technologies, San Diego, CA, USA) [22] 6.5-digital multimeter operating as voltmeter—this setup enables the use of the measurements from the multimeter or from the calibrator to assess the uncertainties of the sensor-box input. An Agilent 3458A 8.5-digit multimeter (Keysight Technologies Inc., Santa Rosa, CA, USA) [23], also operating as voltmeter, measures the sensor-box output voltage. Both multimeters are also controlled by IEEE 488.2 to collect their AC measurements.

In accordance with the GUM [7] recommendations for Type B uncertainties derived from instrument specifications, the probability density functions (PDFs) of the instruments’ measured values are considered to be uniform, centered on the measured values, and the uniform PDF width is twice the maximum error obtained from the instrument specifications [21,22,23].

A set of sensor input/output measurements were obtained, with input RMS voltage in the 4 V to 300 V range, to estimate the sensor-box straight-line parameters. For each measured value the maximum error was determined using the specifications of the instruments [21,22,23]—notice that the maximum errors (and uncertainties), as specified by the manufacturers, depend on the ranges used for each measured value.

The complete set of measured data and the corresponding maximum errors are available as detailed in the Data Availability Statement.

## 4. Results

This section presents the study’s principal findings, beginning with calibration examples that illustrate the practical application of the MCM, followed by a performance evaluation of the method under varying measurement conditions, and concluding with an analysis of the dispersion and correlation of the estimated parameters.

### 4.1. Calibration Examples

**Figure 5:**
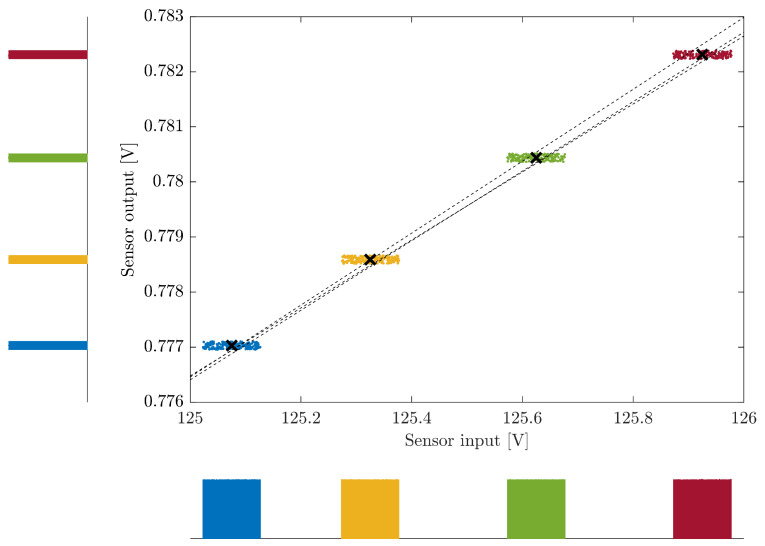
Example of the proposed MCM-based calibration method using only four of the measured values in the 125 V to 126 V sensor-box input range. The crosses represent the measured input/output values, and for each of the four values, 200 realizations of the MCM are shown. Also shown are three examples of the resulting regression straight-lines. The sensor-box input values/uncertainties used correspond to the Wavetek 9100 calibrator. The histograms obtained with $10^{7}$ realizations of the MCM sensor input and output are shown near both axes.

To demonstrate the operation of the MCM, a small subset of the measurements was used. Four measured pairs were selected in a small range of the input voltage, between 125 V and 126 V, and the corresponding results are depicted in Figure 5 using the Wavetek 9100 for the measurements/uncertainties of the sensor-box input. The crosses represent the measured values and around these, 200 realizations of the MCM are represented for each pair considering the uncertainties of both input and output voltages. Notice that this reduced value of realizations was selected to enable visualization of the process, as it is typical that the number of MCM realizations vastly exceeds this value. Also depicted are three of the resulting best fit straight-lines. For these sensor-box input values, the calibrator maximum errors are approximately 52 mV (which corresponds to about 0.04 %), while the maximum sensor-box output errors (from the Agilent 3458A) are approximately 74.5 μV (about 0.01 %).

**Figure 6:**
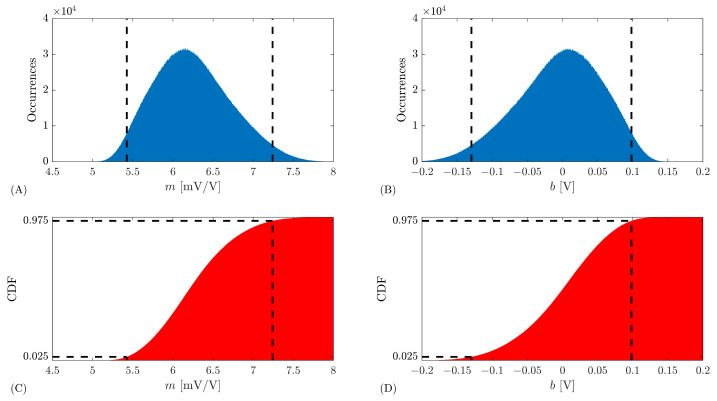
MCM histogram for the regression slope *m* (**A**) and for the regression offset *b* (**B**). The corresponding CDFs are shown in (**C**,**D**). These results were obtained for the example with four measurements from Figure 5 with $M=10^{7}$ realizations of the MCM.

The estimated PDFs obtained from the histogram with $M=10^{7}$ repetitions of the regression slope *m* and offset *b* parameters are shown in Figure 6A,B, with 1000 bins. Notice that the histograms are not Gaussian, presenting a skew. To validate this, a chi-square goodness of fit test for Gaussian distribution was performed on the histograms of Figure 6, which resulted in a $\chi_{\mathrm{obs}}^{2}=3.28\times 10^{5}$ for both *m* and *b*, with ν=997 degrees of freedom. The value of $\chi_{\mathrm{obs}}^{2}$ exceeds the 95 % quantile of $\chi_{\nu }^{2}$, which is $1.07\times 10^{3}$. Therefore, there is reason to conclude that *m* and *b* PDFs are not normally distributed.

The corresponding estimated CDFs are depicted in Figure 6C,D together with the limits that define the interval estimated to have a 95 % level of confidence obtained from the CDF interceptions with 2.5 % and 97.5 %. The resulting interval for the regression slope *m* is $\left[5.43;\;7.24\right]\;\mathrm{mV}/V$ and $\left[-0.13;\;0.10\right]\;V$ for the regression offset *b*. The uncertainty, corresponding to a 95 % level of confidence, $U_{95,\mathrm{MCM}}$, is the half-width of these intervals. For the regression slope, $U_{95,\mathrm{MCM}}\left(m\right)=0.91\;\mathrm{mV}/V$, while for the offset, $U_{95,\mathrm{MCM}}\left(b\right)=0.11\;V$. In this particular case, with only four data pairs for the regression, and positioned very close together, there is a strong correlation between the *m* and *b* estimates with a −0.9999968 correlation coefficient.

In Appendix A, an example considering correlation between the four sensor-box input measurements is presented that also includes correlation between the sensor-box output measurements. This case study highlights the ability of the MCM method to take into account any type of correlations in the measured values.

**Figure 7:**
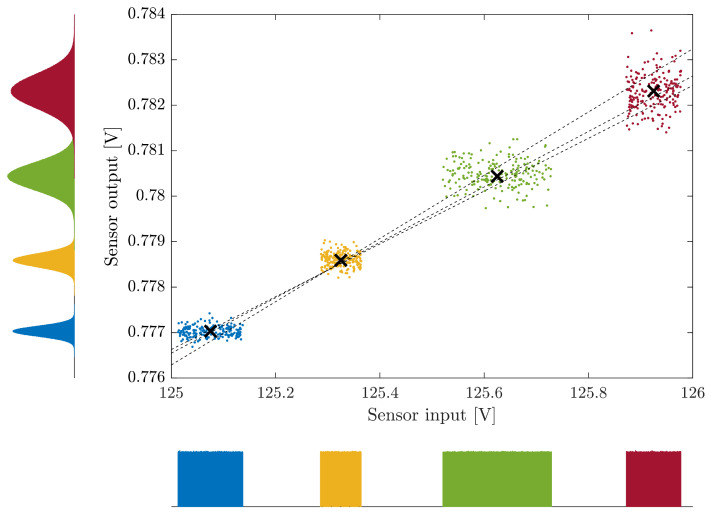
Example of the proposed MCM-based calibration method considering the four measurement pairs from Figure 5 but with altered PDFs for the sensor-box input (uniform PDFs with different uncertainties) and sensor-box output (normal PDFs with different uncertainties). Three examples of the regression line results are shown with black dashed lines. The histograms obtained with $10^{7}$ realizations of the MCM sensor input and output are shown near both axes.

To demonstrate the flexibility of the proposed method, these four measured input/output sensor-box pairs are considered but the actual measurement PDFs are discarded, and alternative PDFs are considered. Uniform PDFs are still used for the sensor-box input measurements, but their width is changed for each value of $x_{i}$. For the sensor-box output measurements, the PDFs were changed to normal/Gaussian PDFs and their standard deviations were also modified for each $y_{i}$. The results for 200 realizations and three possible regression straight-lines are shown in Figure 7. The normal PDFs in the sensor output and their increased uncertainty result in some outliers that were not present on the real sensor-box output values (Figure 5) due to their uniform PDFs.

### 4.2. Performance Evaluation

To evaluate the proposed method performance, the number of measurement pairs considered for the calibration process was increased from N=2 up to $N=10^{4}$ with equally spaced values for each case. Within the measurement range, the first and last measured pairs of values are always used and the MCM was used to estimate the statistics of the regression coefficients.

**Figure 8:**
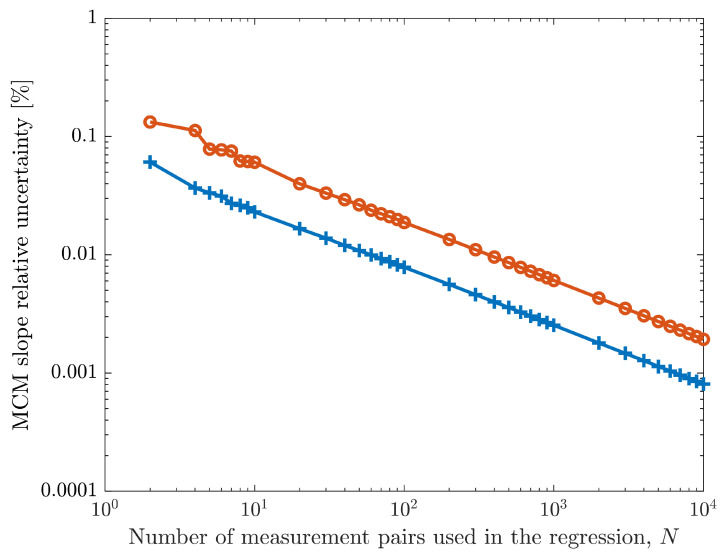
MCM relative uncertainty of the calibration slope *m* as a function of the number of measurement pairs used in the calibration. The results in blue were obtained using the $x_{i}$ values and corresponding uncertainties from the Wavetek 9100 calibrator. The results in red use the measurements and uncertainties from the Agilent 34410A, which is measuring the sensor-box input. The measurements always include the first and last sensor-box pairs of values and are equally spaced within this range.

The relative uncertainty of the regression slope, $U_{95,\mathrm{MCM}}\left(m\right)$, obtained from the CDF following the process exemplified in Figure 6, as a function of the number of pairs, *N*, used in the regression is shown in Figure 8. Two different situations are represented in this figure. The red line represents the results when the Agilent 34410A measurements/uncertainties are considered for the sensor-box input values. The blue line represents the results obtained when the Wavetek 9100 measurements and uncertainties are considered as sensor-box inputs.

As expected, the slope relative uncertainty decreases with increased number of regression points, and the relative uncertainty is lower when the calibrator values of the sensor-box input are used—the relation between the two maximum errors/uncertainties is not constant due to the different range changes, but the ratio is almost always above 2 and can reach, for some values, almost 10.

**Figure 9:**
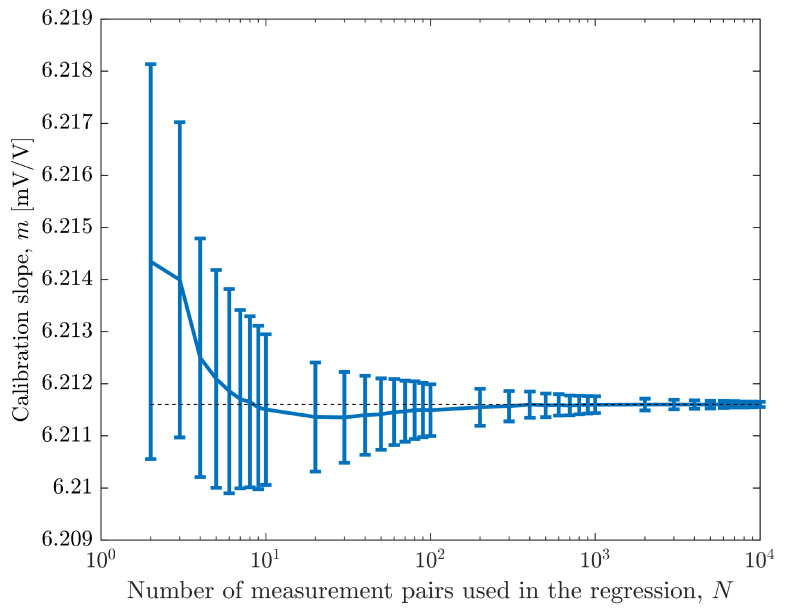
Average value of the calibration slope *m* and corresponding interval estimated to have a 95 % level of confidence (shown as error bars) as a function of the number of measurement pairs, *N*, used in the MCM. These results were obtained using always the first and last sensor-box input values, with equally spaced measurements and using the calibrator measurements/uncertainties. The dashed line is the average value when $N=10^{4}$ measurement pairs are used for the regression.

The average value of the calibration slope *m* and the corresponding intervals obtained for a 95 % level of confidence as a function of the number of measurement pairs used in the MCM method are shown in Figure 9. These results were obtained using the calibrator measurements/uncertainties and show the reduction of the interval and convergence to the center value, represented by the horizontal dashed line, that was obtained for $N=10^{4}$ measurement pairs.

Table 1 presents some of the numerical results from Figure 9, which show that a 100-fold increase in the number of regression values reduces the relative uncertainty of the slope by 10. This is also observed in the results presented in Figure 8 (blue line) and is also valid for the results obtained with the Agilent 34410A instrument (red line in Figure 8). The values in the last column in Table 1 are the expanded uncertainty corresponding to a 95 % level of confidence using the same measurements with the WTLS algorithm, $U_{95,\mathrm{WTLS}}$. Notice that these values converge to the uncertainties of the MCM (second column) as the number of regression measurement pairs increase.

**Figure 10:**
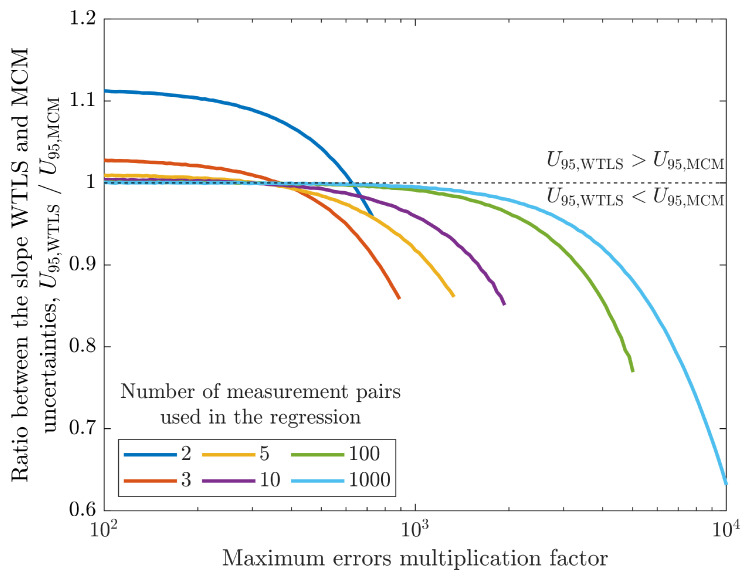
Ratio between the slope expanded uncertainty obtained with the WTLS method $\left(U_{95,\mathrm{WTLS}}\right)$ and the MCM uncertainty $\left(U_{95,\mathrm{MCM}}\right)$ as a function of the artificial factor with which the measurement maximum errors are increased in relation to the actual maximum errors. The results are presented for six different values of measurement pairs (2, 3, 5, 10, 100 and 1000).

To analyze the influence of the maximum measurement errors on the WTLS and MCM, these were artificially increased by a multiplication factor and the results obtained with both methods were registered. Figure 10 shows the ratio between the slope uncertainties of both methods as the maximum error multiplication factor is increased up to $10^{4}$. The figure shows how this ratio changes for situations with different numbers of processed measurement pairs (2, 3, 5, 10, 100 and 1000). Results are only shown until the relative MCM uncertainty reaches 50 % since for larger errors the estimated slope is no longer relevant due to its large uncertainty. The results show that the WTLS expanded uncertainty $\left(U_{95,\mathrm{WTLS}}\right)$ is over-estimated (i.e., it is larger than $U_{95,\mathrm{MCM}}$) when the regression is done with few measurement pairs and until a certain level of measurement errors, after which it becomes significantly under-estimated. When there is a significant number of regression pairs, both methods present identical uncertainties, but for larger measurement errors the WTLS uncertainty is significantly under-estimated. These two factors combined show that there are significant limitations to the validity of the WTLS estimated uncertainty that depend on the number of measurements used in the regression and on the level of measurement maximum errors.

### 4.3. Dispersion and Correlation Analysis

**Figure 11:**
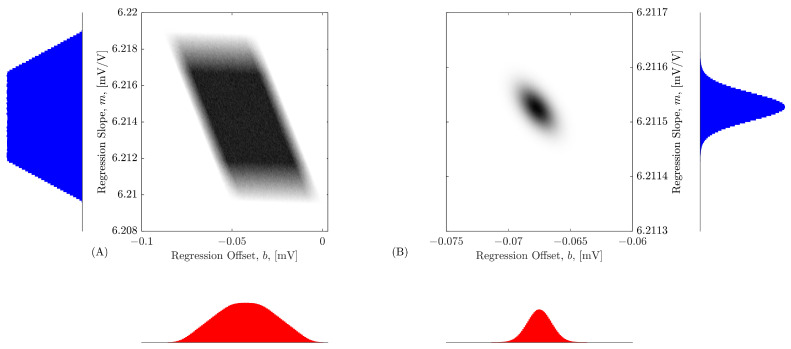
Intensity plot of the occurrences showing the correlation between the estimated straight-line slope *m* and offset *b*. The results in (**A**) correspond to the use of only two pairs of measured values in the regression (the first with $V_{1}=4\;V$ and the last with $V_{1}=300\;V$). The results shown in (**B**) were obtained using $N=10^{4}$ uniformly distributed measured pairs of values in the regression. The blue histograms represent the regression slope of both cases and the red histograms represent the regression offsets.

The intensity plots shown in Figure 11 highlight the MCM-based regression method’s ability to show the dispersion of estimated regression slope *m*, offset *b* and their correlation. The results in Figure 11A correspond to the regression when only the two sensor-box input extreme measurement pairs are used. There is, as expected, a large dispersion of the estimated values, and the correlation coefficient is −0.565. The chi-square goodness of fit test for Gaussian distribution results in $\chi_{\mathrm{obs}}^{2}=1.06\times 10^{6}$ for *m* and $\chi_{\mathrm{obs}}^{2}=2.79\times 10^{5}$ for *b*, with ν=197 degrees of freedom. Both values of $\chi_{\mathrm{obs}}^{2}$ exceed the 95 % quantile of $\chi_{\nu }^{2}=231$, leading to the conclusion that, when 2 measurement pairs are used for the regression, *m* and *b* values are not normally distributed. Using a total of $N=10^{4}$ uniformly spaced measurement pairs for the regression results in the intensity plot depicted in Figure 11B. The results occupy a smaller area as a result of the better estimates obtained in this case, and the correlation coefficient is −0.562. Additionally, the chi-square goodness of fit test for Gaussian distribution results in $\chi_{\mathrm{obs}}^{2}=210$ for *m* and $\chi_{\mathrm{obs}}^{2}=205$ for *b*, with ν=197 degrees of freedom. Since the 95 % quantile of $\chi_{\nu }^{2}$ is 231, it cannot be excluded that, when $10^{4}$ measurement pairs are used for the regression, *m* and *b* values are normally distributed.

## 5. Selection Strategy of Calibration Points

One important aspect that must be considered when performing sensor calibration is the selection of which sensor input values to measure. The relevance of this decision is derived by three issues related with increasing the number of measured values: (i) it usually results in higher costs—for example, a liquid viscosity sensor [24] requires the availability of multiple costly solutions with different viscosities; (ii) it requires a lengthier calibration process—in the voltage sensor-box example, increasing the number of input voltages requires changing the calibrator voltage, waiting a specified amount of time to stabilize the sensor input and output voltages before executing the measurements; (iii) finally, what is the desired uncertainty of the calibration coefficients. Within this context it is of crucial importance to minimize the number of measured values in the calibration process. This section aims to discuss how to choose the sensor input values to optimize the regression slope estimation uncertainty.

**Figure 12:**
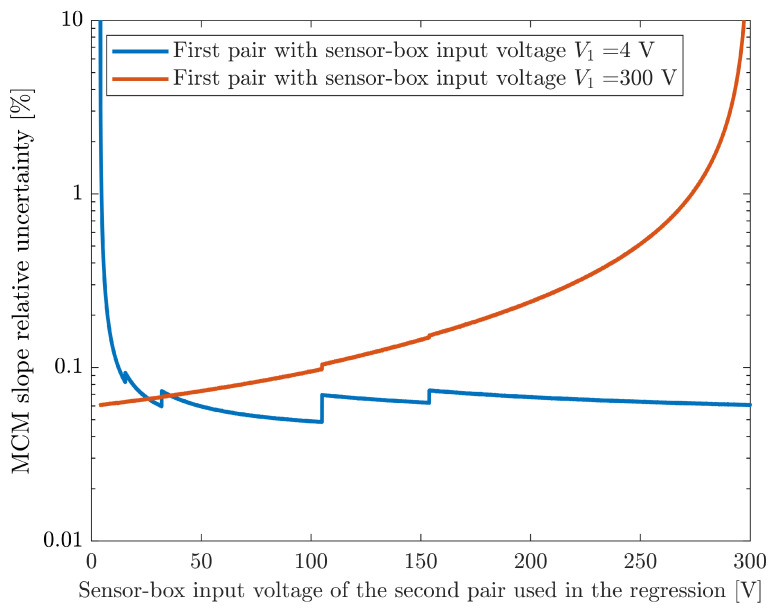
MCM slope relative uncertainty when using only two pairs of measured values. The red line results correspond to always using the last measured pair (which has $V_{1}=300\;V$), while the blue line results always use the first measured pair (which has $V_{1}=4\;V$).

The first analysis includes using only two pairs of measured values, which corresponds to the minimum number of pairs that can be used to estimate the sensor calibration coefficients. The results presented in Figure 12 correspond to the relative uncertainty of the slope regression estimates, with the red line corresponding to the situation where the last measured pair (with $V_{1}=300\;V$) is used and the influence of the second pair input voltage is analyzed. As expected, the lowest uncertainty is obtained when the second value is furthest apart, i.e., when the second value is the first measured pair (with $V_{1}=4\;V$). Notice the very large uncertainty (nearly 10 %) when the second value is near the last pair. In this case, the two pairs used in the regression are too close and this results in a very large uncertainty in the estimated slope. There is a small discontinuity near the pair with $V_{1}=105\;V$ due to a change in the Wavetek 9100 calibrator range, which provides the sensor-box input voltage, $V_{1}$ (it switches from the 105 V to the 320 V range).

The results presented in the blue line of Figure 12 correspond to the situation where the first measured pair is always used ($V_{1}=4\;V$) and the second pair used in the regression is changed. In this case, there are four discontinuities near $V_{1}=15\;V$, $V_{1}=32\;V$, $V_{1}=105\;V$ and $V_{1}=155\;V$. These discontinuities are caused by the different maximum errors associated with the ranges of the two instruments. The first (near $V_{1}=15\;V$) and last (near $V_{1}=155\;V$) discontinuities are caused by the Agilent 3458A (which is measuring the sensor-box output) changing ranges from 0.1V to 1V and then from 1V to 10V. The others are caused by the range change from the Wavetek 9100 calibrator (which corresponds to the sensor-box input) from 32V to 105V and from 105V to 320V. It should be noted that these discontinuities are also present in the red line of Figure 12 but are not as noticeable. The last noteworthy aspect of these results is that the lowest slope uncertainty is not obtained using the two most extreme measured pairs of values but instead using $V_{1}=4\;V$ and $V_{1}=104.975\;V$—which is immediately before the Wavetek calibrator switches from the 105 V to the 320 V range. This is because it has a lower sensor-box input relative uncertainty than the $V_{1}=300\;V$ value (approximately 0.046 % for $V_{1}=104.975\;V$ compared with 0.056 % for $V_{1}=300\;V$).

**Figure 13:**
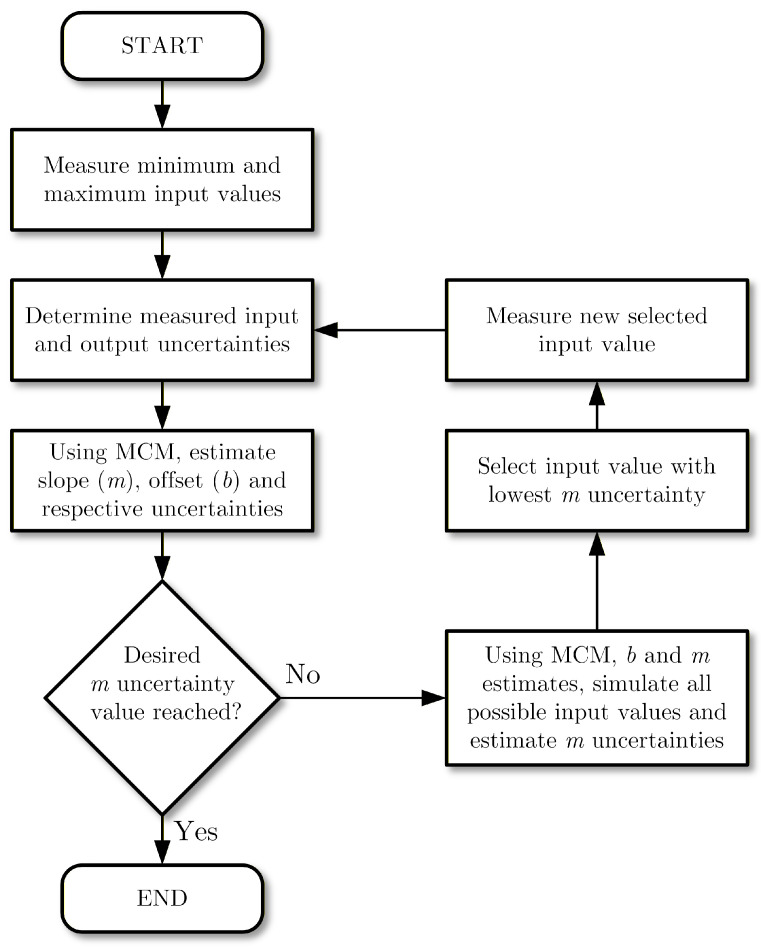
Flowchart of the optimization procedure to iteratively select each new input value to be measured in order to improve the estimated slope uncertainty until a pre-defined threshold is reached.

The number of calibration points and their selection can be optimized using the MCM method by simulating, before measuring, the impact of possible new input values on the uncertainty of the estimated parameters. A possible optimization process is proposed and detailed in the flowchart presented in Figure 13. The process starts with measuring the two most extreme input values and determining, from these measurements, their uncertainties. The next step is to estimate the slope and offset calibration coefficients and their uncertainties using the MCM. At this stage, the slope uncertainty is compared with the desired uncertainty for this parameter—if the current value has reached the desired target, the calibration is stopped (notice that this description is aimed solely for the slope parameter but can be adapted to include the offset as well). If the target uncertainty has not been reached, the process will evaluate each possible input value (added to the already performed measurements) simulating the output values using the updated slope and offset parameters. From the set of simulated input values, the one that produced the smallest slope uncertainty is selected to be measured. The process is then repeated with the inclusion of the new measurement pair. It should be noted that a limit on the number of new measurements can be added to this iterative process as well as a condition to stop if the slope uncertainty does not improve significantly.

**Figure 14:**
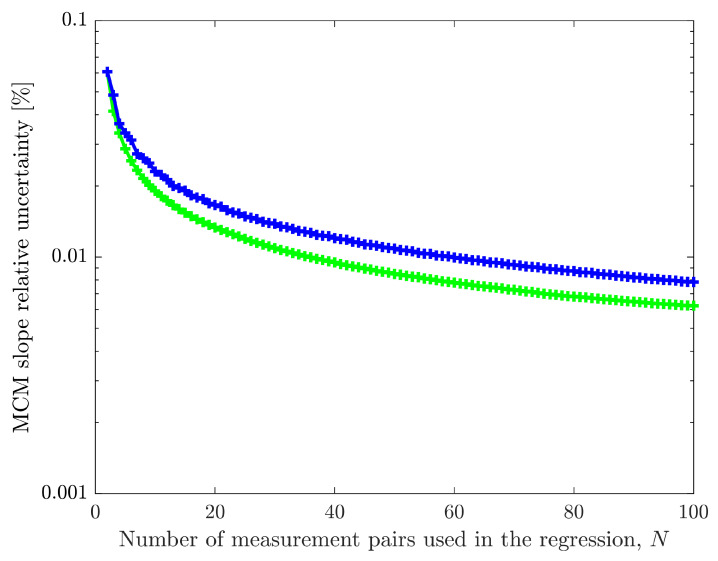
Comparison between the MCM obtained slope relative uncertainty as a function of the number of measurements using equally spaced input measurements (blue line) and the proposed optimization process (green line) described in the flowchart of Figure 13.

The green line in Figure 14 shows the results obtained with the optimization process. The non-optimized selection of evenly spaced input values is presented in the blue line (this corresponds to the blue line from Figure 8). The results when using only two measurements are the same but as the number of measurements increases, the uncertainties obtained with the optimization process are lower, thus highlighting the advantage of the optimization process. A notable case is the selection of the third measurement to be performed. While the equally spaced process uses 152 V (half-way between 4 V and 300 V), the proposed optimization selects 104.975 V, which is the value immediately before the calibrator switches ranges, as described in the analysis of the blue line in Figure 12. Although the differences may seem only incremental, they can, in some cases, present significant improvements. For example, to achieve a 0.01 % slope relative uncertainty target, the equal spacing option (blue line) requires 60 measurement pairs, while the proposed optimization process (green line) requires only 37.

## 6. Conclusions

This paper presents an analysis of sensor calibration using the Monte Carlo Method (MCM), specifically for scenarios where the sensor input/output relation is modeled by a straight-line relationship. Traditional regression techniques include Least Squares (LS), Total Least Squares (TLS), Deming, and Weighted Total Least Squares (WTLS). These methods have limitations in handling measurement uncertainties, especially when they include sensor input and output measurements with non-Gaussian distributions and/or covariances. The Monte Carlo-based approach provides a flexible and robust alternative that accounts for heteroscedastic measurement uncertainties, non-Gaussian distributions, and PDF propagation including input covariances.

Through an experimental setup using a Hall-effect voltage sensor-box, the method was validated and shown to yield accurate estimates of regression parameters along with statistically sound intervals with a 95 % level of confidence, as shown in Figure 9. The presented results show that (i) MCM allows the use of any measurement PDFs for both input and output variables; (ii) for the presented example, increasing the number of calibration points reduces the estimation uncertainties, with the relative uncertainty in the slope decreasing approximately with the square root of the number of measured points; (iii) the location and uncertainty characteristics of selected measurement points significantly affects the uncertainty of the calibration coefficients, particularly when using a limited number of points; (iv) the method can be used to assess the correlation of the estimated calibration coefficients, making it ideal for sensor systems operating under diverse real conditions. Overall, the Monte Carlo method is a versatile tool for sensor calibration, particularly in applications that require rigorous uncertainty evaluation. This approach ensures compliance with current metrological guidelines and can be used in a wide range of sensor types and measurement conditions.

An optimization process to select the ideal input measurements is proposed. It uses MCM on simulated calibration points to evaluate which input value should be measured to minimize the slope uncertainty and it is used until a target slope uncertainty is reached. In the described example, for a target relative uncertainty of 0.01 % in the slope parameter, the optimized process required 37 measurement pairs, while using equally spaced measurements needed 60. This result demonstrates that the process can be used to improve cost and time efficiency in sensor calibration.

While the presented example did not incorporate sensor input covariances, which could affect calibration accuracy in systems where such correlations are significant, the Monte Carlo method is capable of handling correlated input measurements.

Future work will focus on extending the Monte Carlo-based calibration approach to nonlinear sensor models and evaluating its performance across a broader range of sensor types and uncertainty distributions.

## Figures and Tables

**Table 1:** Regression slope uncertainty and relative uncertainty for an interval estimated to have a 95 % level of confidence, as a function of the number of measurement pairs used in the regression. These results correspond to the situation where the Wavetek 9100 calibrator measurements/uncertainties were used as the sensor-box input (Figure 9). The last column reports the expanded uncertainty to have a 95 % level of confidence obtained by using the WTLS for the same set of measurement pairs.

Number of Measurement Pairs Used in the Regression	Slope MCM Uncertainty $U_{95,\mathrm{MCM}}\left(m\right)$ [µV/V]	Slope MCM Relative Uncertainty [%]	Slope MCM Interval Estimated to Have a 95 % Level of Confidence [mV/V]	Slope WTLS Expanded Uncertainty $U_{95,\mathrm{WTLS}}\left(m\right)$ [µV/V]
2	3.8	0.061	[6.2105; 6.2181]	4.2
3	3.0	0.048	[6.2109; 6.2169]	3.1
4	2.3	0.037	[6.2102; 6.2147]	2.3
5	2.1	0.033	[6.2100; 6.2141]	2.1
10	1.4	0.023	[6.2100; 6.2129]	1.4
100	0.49	0.0079	[6.21094; 6.21192]	0.49
1000	0.16	0.0025	[6.21136; 6.21168]	0.16
10 000	0.050	0.00081	[6.211478; 6.211578]	0.050

**Figure A1:**
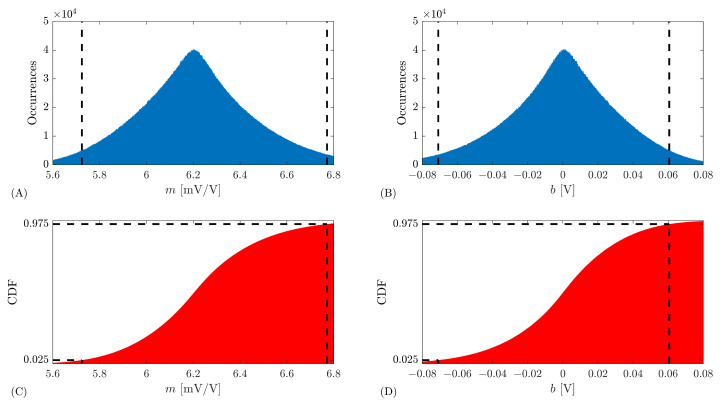
MCM histogram for the regression slope *m* (**A**) and for the regression offset *b* (**B**). The corresponding CDFs are shown in (**C**,**D**). These results were obtained for the example with four measurements from Figure 5 with $M=10^{7}$ realizations of the MCM but now considering a 0.7 correlation between the sensor-box input voltage measurements as well as the same correlation factor between the output voltage measurements.

## Data Availability

Dataset is available at https://github.com/pedro-m-ramos/voltage-sensor-box-measurements (accessed on 22 April 2026).

## Appendix A

This appendix presents an example application of the MCM where correlations are considered. The example is based on the four-pair measurement situations from Figure 5 and Figure 6. For this particular case-study a correlation of 0.7 is included between the four measured values of the sensor-box input voltage and also between the four sensor-box output voltages. The individual PDFs remain uniform, as depicted in Figure 5. The results presented here are not an in-depth analysis of the effects of the correlation but instead demonstrate the ability of the MCM to take into account these correlations. The interval estimated to have a 95 % level of confidence obtained from the CDF for the regression slope *m* is $\left[5.72;\;6.77\right]\;\mathrm{mV}/V$ and $\left[-0.071;\;0.061\right]\;V$ for the regression offset *b*. For the regression slope, $U_{95,\mathrm{MCM}}\left(m\right)=0.52\;\mathrm{mV}/V$, while for the offset $U_{95,\mathrm{MCM}}\left(b\right)=0.066\;V$. The WTLS, which does not consider correlations, estimates a slope expanded uncertainty of $U_{95,\mathrm{WTLS}}\left(m\right)=0.91$ mV/V and an offset expanded uncertainty of $U_{95,\mathrm{WTLS}}\left(b\right)=0.11$ V. These values are very close to the ones estimated by the MCM when no correlation was considered but are significantly different from the MCM values when the correlation is included.
